# Cosmetic colouring by Bearded Vultures *Gypaetus barbatus*: still no evidence for an antibacterial function

**DOI:** 10.7717/peerj.6783

**Published:** 2019-05-15

**Authors:** Antoni Margalida, Markus S. Braun, Juan José Negro, Karl Schulze-Hagen, Michael Wink

**Affiliations:** 1 Institute for Game and Wildlife Research, IREC (CSIC-UCLM-JCCM), Ciudad Real, Spain; 2 Heidelberg University, Institute of Pharmacy and Molecular Biotechnology, Heidelberg, Germany; 3 Estación Biológica de Doñana (CSIC), Sevilla, Spain; 4 Moenchengladbach, Germany; 5 University of Bern, Division of Conservation Biology, Bern, Switzerland

**Keywords:** Breeding success, *Gypaetus barbatus*, Iron oxides, Antibacterial hypothesis, Status signalling

## Abstract

Bearded Vultures regularly visit ferruginous springs for cosmetic purposes to obtain their reddish plumage colouration. Different hypotheses have been proposed to explain this deliberate application of adventitious colouration: (1) to signal individual dominance status; (2) to exploit an anti-bacterial effect of iron oxides or ochre to reduce feather degradation by bacteria and, in parallel (3) to enable incubating birds to transfer this protection to their developing embryos to increase hatching success. Here, we re-evaluate the antibacterial hypothesis using three experimental approaches: (a) by applying feather-degrading bacteria to stained and unstained bearded vulture feathers; (b) by assessing the antibacterial activity of ochre; and (c) by comparing the breeding success of orange individuals with pale ones. Our findings suggest that the *in vitro* addition of feather degrading *Bacillus licheniformis* to naturally stained Bearded Vulture feathers did not retard feather degradation compared to controls. Iron particles from red soil (ochre) or iron salts had no antibacterial effect on the growth of three species of bacteria (*Escherichia coli, Kocuria rhizophila* and *Bacillus licheniformis*), incubated either in the dark or under visible light. Finally, breeding success did not differ between territories occupied by pale individuals versus orange ones. These results run counter to the hypothesis that iron oxides have an antibacterial role in Bearded Vultures. The use of red soils by Bearded Vultures may function as a territorial status signal, but may also be involved in other processes, such as pair formation and the long-term maintenance of the pair bond, as suggested for the closely related Egyptian vulture *Neophron percnopterus*.

## Introduction

Bearded Vultures (*Gypaetus barbatus*), along with the closely related Egyptian Vulture (*Neophron percnopterus*) (Wink, 1995; Lerner & Mindell, 2005), are unique among raptors in applying cosmetic colouration to their plumage (Negro & Margalida, 2000; Van Overveld, de la Riva & Donázar, 2017). Cosmetic colouration has been defined as the active and deliberate application of adventitious (external) pigments by a bird to its plumage, and has been reported in at least 28 bird species (Delhey, Peters & Kempenaers, 2007). These pigments may be secreted by the birds themselves through the uropygial gland (Amat et al., 2011) and the skin, or obtained externally from geological substrates. The bright red-orange colouration on the neck, head and ventral parts of adult Bearded Vultures is due to iron-oxide particles obtained from red soils usually known as ochre (Brown & Bruton, 1991; Houston, Hall & Frey, 1993; Negro & Margalida, 2000). The first suggestion that the rufous colour of Bearded Vultures’ plumage derives from iron-oxides obtained from ferruginous waters was discussed as long ago as the 1870s (Meves, 1875) and mentioned again 50 years ago (Berthold, 1967).

**Figure 1:**
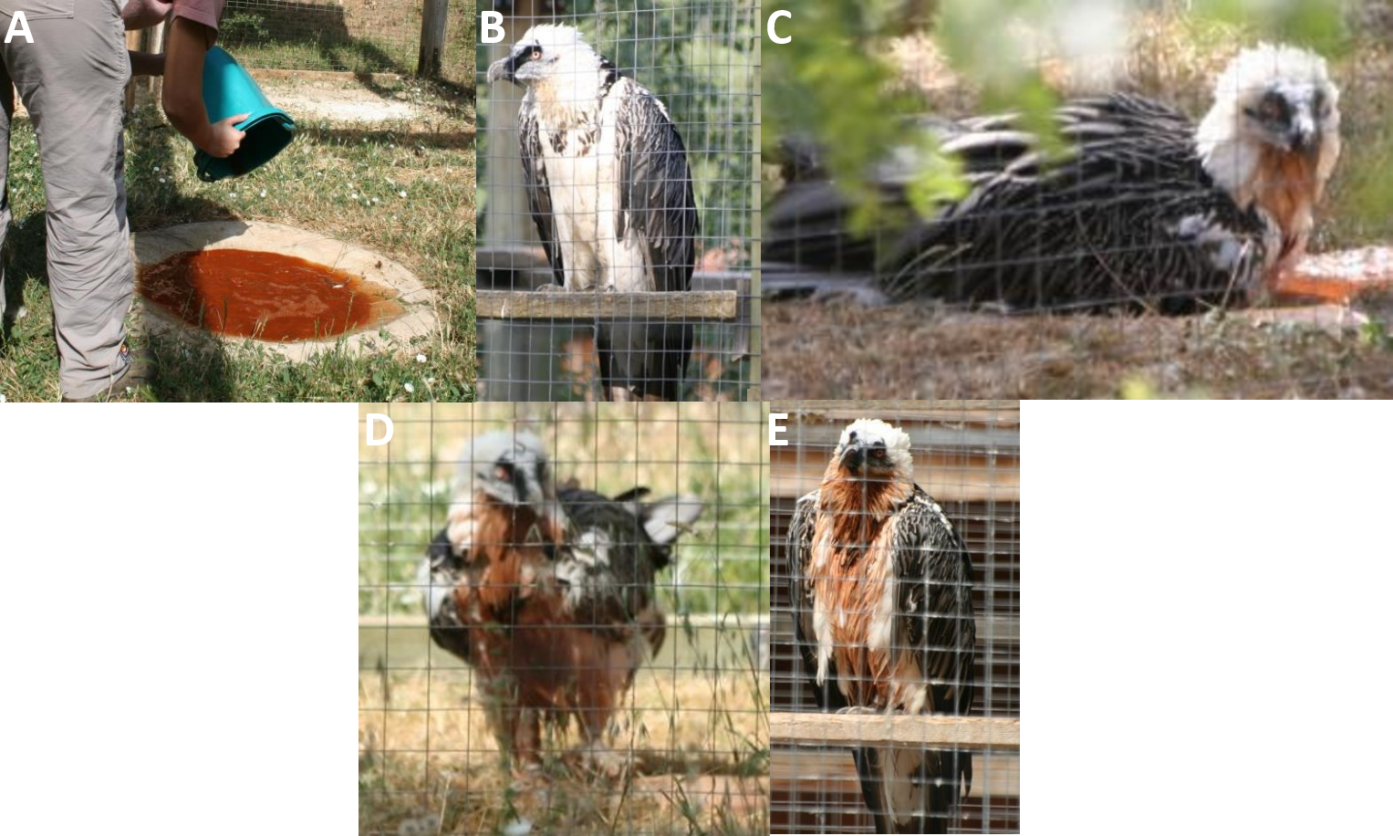
Experimental observations of Bearded Vulture bathing behaviour at the Guadalentín recovery center. (A) The ferruginous water is prepared; (B) a completely white adult female perches nearby; (C–D) the adult female takes a bath; and (E) perches again after the bath.

The debate as to whether this cosmetic colouration was acquired passively from stained rock ledges or deliberately through bathing in ferruginous waters remained unresolved until very recently. Several authors considered that the colour was acquired accidentally because nobody had actually observed Bearded Vultures bathing in ochreous waters (A. Hume in Meves, 1875; Brown & Bruton, 1991). They believed that the staining was acquired passively and accidentally when vultures came into contact with iron oxide deposits at perching or nesting sites on rocky outcrops. However, an experiment showed that captive birds actively anointed themselves with wet soils, and that when offered wet soils of various colours birds of both sexes and different age classes consistently preferred red soils containing iron oxides (Frey & Roth-Callies, 1994). More recently, free-ranging Bearded Vultures have finally been observed bathing naturally in ferruginous waters containing iron oxides, and thereby actively dying their plumage (Caussimont, Hunot & Mariette, 1995; Margalida & Pelayo, 1998). Subsequently, two of us (JJ Negro and A Margalida, pers. obs., 2004) offered red baths to 16 captive Bearded Vultures kept at a breeding facility in southern Spain (http://www.gypaetus.org). They had been deprived of red soils for several years, but previously had permanent access to clean water baths. All of them bathed in the newly provided ferruginous baths within the first few hours, ignoring the clean water baths for weeks afterwards. They all stained their feathers (ventral parts and neck) with a rich orange tinge, including immature birds with their fully melanised plumages (Fig. 1).

We now know for sure that wild Bearded Vultures regularly make use of ferruginous springs for staining purposes. This type of water can only be found in areas with particular mineralogical, hydrogeological and climatic characteristics (Schwertmann & Taylor, 1989). If no ferruginous waters are available in their territories, adult Bearded Vultures cannot stain themselves and appear white on the ventral areas, e.g., many individuals on the islands of Crete and Corsica (A Margalida & JJ Negro, pers. obs., 2001).

Three hypotheses have been proposed to explain the deliberate application of adventitious colouration: hypothesis 1 is that it signals individual dominance status (Negro et al., 1999; an alternative hypothesis 2 Arlettaz et al., 2002) proposes that iron oxides might have some anti-bacterial and antiparasitic properties; and hypothesis 3 suggests that incubating Bearded Vultures could transfer this protection to their developing embryos (Arlettaz et al., 2002). The antiparasitic part of hypothesis 2 has in fact been tested previously. Frey & Roth-Callies (1994) observed that feather-chewing mallophaga were not affected by iron oxide, reporting that feather degradation was not significantly different in white or stained feathers. In addition, Negro et al. (2002) questioned the toxicity of iron oxide to feather ectoparasites because the mechanism of toxicity was merely hypothetical. Regarding a bacterial and/or antiparasitic attack, iron is an essential element for almost all living organisms, including bacteria, which require it for growth and replication (Miller & Britigan, 1997). Given that bacteria actively seek iron, it is unlikely that they are adversely affected by iron oxides as suggested by Arlettaz et al. (2002). Regarding hypothesis 3, Arlettaz et al. (2002) also suggested that iron oxides may help to mobilise vitamin A, or scavenge free radicals, in the embryo or in nestlings. This hypothesis would require the transfer of iron oxides through the eggshell, or through the chick’s skin, and it has yet to be shown that such transfer is possible.

A recent paper by Tributsch (2016) also suggested that although the potential of iron oxide as an antibacterial agent has been investigated in some detail (i.e., our hypothesis 2), the idea cannot be supported because there is no chemical or biochemical evidence for such activity, and because iron is essential for bacterial metabolism (Negro et al., 2002). However, these previous studies were not aware that an antibacterial activity of iron oxide might require energy from UV irradiation, suggesting that the photochemical aspects of ochre activity in sunlight should be taken into consideration.

Here we performed chemical analysis on red-coloured soil (ochre) from a water bath in the Pyrenean Mountains and we describe the chemical composition of impregnated iron oxide feathers. In order to re-evaluate the antibacterial hypothesis (hypothesis 2) we investigated the antibacterial activity of ochre from a ferruginous spring in the Pyrenees, which we know has been traditionally used by Bearded Vultures, both at night (in the dark) and during the day. We tested hypothesis 3 by looking to see whether the breeding success of territories held by orange individuals is higher than those held by pale individuals. In addition, we use data on feather-degradation by *Bacillus licheniformis* (JJ Negro, pers. obs., 2004). Bearded vulture feathers were tested along with those of other bird species for which the results of the experiment are already available (Grande, Negro & Torres, 2004).

## Material and Methods

The study area lies within the Pyrenean Axial Zone (NW Catalonia, Spain). Its climate is typical of the Pyrenean valleys, with a regular pattern of annual rainfall exceeding 1,000 mm a year. The annual average temperature does not oscillate significantly and varies between 9–10.5 °C, with a high in August (29 °C) and a low in January (−3 °C). The field work was carried out under the recovery plan of the bearded vulture in Catalonia (Departament de Medi Ambient i Habitatge from Generalitat de Catalunya, project 1992–2006).

**Figure 2:**
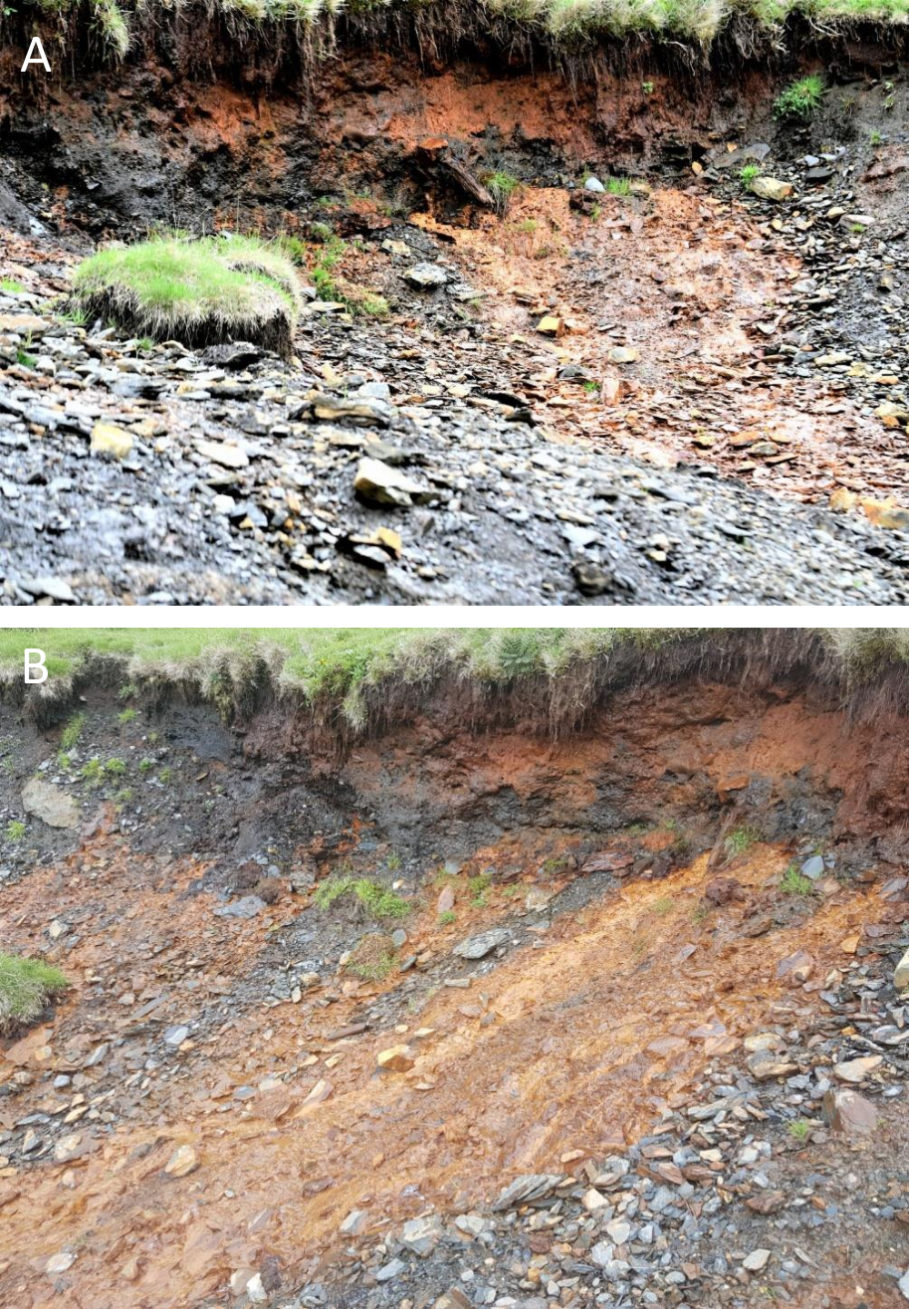
Detail of ferruginous waters. Leaching of iron oxides from soil into the water (A). The site is frequented by Bearded Vultures (B). Photos by Michael Wink.

Bearded Vultures regularly visit places rich in ochre deposits and red-coloured waters, where they impregnate their white feathers, which become orange as a consequence. A known ochre-rich bathing site, frequented by Bearded Vultures (Fig. 2), is situated near a breeding site (3.7 km distant) at 1,450 m above sea level.

### Feather analyses

We collected feathers of Bearded Vultures found near an ochre bath and at adjacent sites (Margalida & Pelayo, 1998) for analysis using a scanning electronic microscope (SEM). Seven feathers coloured with iron oxides from three different territories were mounted on a stub and coated with carbon to ensure a conductive surface. The SEM morphological and analytical feather images were produced using a Jeol JSM 840, which allowed imaging and a semi-quantitative microanalysis of mineral elements on the feathers. In addition to the morphology, it was possible to observe the density contrast of the different metallic surfaces based on discrimination of the backscattered elements.

### Feather-degrading bacteria and iron oxides (hypothesis 2)

The degradation of feathers from selected species containing different natural pigments has been described previously by Grande, Negro & Torres (2004). It has been hypothesized that iron oxides applied externally would protect Bearded Vulture feathers from feather-degrading bacteria (Arlettaz et al., 2002; Tributsch, 2016) in addition to any protection normally afforded by pigments naturally present in feathers. The effects of *Bacillus licheniformis* applied *in vitro* were tested on Bearded Vulture covert and body feathers of different original colours covered or not by iron oxides, as explained in Table 1. The methods used for exposing these feathers to feather-degrading Bacilli, and to assess the level of degradation with time, were described in Grande, Negro & Torres (2004), as the vulture feathers were in fact part of the same experimental setting. The data corresponding to the bearded vultures were never published because their samples were collected in the wild as shed feathers, and might have been exposed to feather-degrading agents. The material from the remaining species was obtained from museum specimens. One of us (JJN) participated in the aforementioned study and was one of the co-authors. Given that the results reported in Grande, Negro & Torres (2004) were obtained using the same bacterial strains (*Bacillus licheniformis* CMP L5, isolated from soil in California, USA), as well as same culture media and culture conditions , we will discuss our results in relation to those previously reported for feathers of other species containing different pigments.

**Table 1:** Results on feather-degradation time by *Bacillus licheniformis* (applied *in vitro*) and tested on Bearded Vulture covert and body feathers of different original colours, covered or not by iron oxides.

**Feather number**	**Basal color**	**Stained**	**Days to full degradation**
1	White	yes	7
2	Part white/part dark	no	7
3	Part white/part dark	no	7
4	Dark	yes	7
5	Dark	yes	7
6	Dark	no	7
7	Dark	no	7
8	White	yes	7
9	White	yes	7

### Antimicrobial activity of ochre and iron oxides: Medium and culture conditions (hypothesis 2)

The antimicrobial activities of iron salts, and of ochre, collected from Bearded Vulture bathing sites, were investigated under light and dark conditions using *Escherichia coli* XL1-Blue MRF‘ (Gram-negative) and *Kocuria rhizophila* DSM 11926^T^(Gram-positive). In addition, the keratinolytic *Bacillus licheniformis* DSM 13^T^ was used to assess the effects of ochre on feather-degrading microorganisms. This strain of *Bacillus licheniformis* was different to the one used for the degradation experiment on feathers described above (Grande, Negro & Torres, 2004). All bacteria were obtained from the German Collection of Microorganisms and Cell Cultures (DSMZ, Braunschweig, Germany), except for *E. coli* which was purchased from Stratagene (San Diego, CA, USA). All bacteria were maintained on Müller-Hinton Agar (MHA) at optimum temperatures, and fresh cultures were used for each test.

### Suspension tests (hypothesis 2)

Suspension tests were conducted to evaluate the antimicrobial potential of ochre and iron oxides when exposed to visible light (16 W, 880 lumen, 140 mA, 2,700 K) compared with dark conditions. The ochre sample was sterilized using dry heat (180 °C for 2 h) and adjusted to 10 mg/mL (w/v) in Müller-Hinton Broth (MHB). Iron(II) oxide and iron(III) oxide (Sigma Aldrich, Steinheim, Germany) were combined at a concentration of 5 mg/mL each in MHB and tested for antimicrobial effects in parallel with the ochre sample.

For the suspension tests, the cell numbers of the indicator strains were adjusted to comparable values using a densitometer (Biosan DEN-1, Biosan Laboratories, Warren, MI, USA). The bacteria were added to the samples to create a final cell density of approximately 5 ×10^5^ cfu/mL. The samples were incubated in liquid media under agitation at 150 rpm at 37 °C. A desk lamp (LM-106B, BAHAG AG, Mannheim, Germany) equipped with a 16 W bulb (Müller-Licht GmbH, Lilienthal, Germany) was used to provide visible light, while the samples to be incubated in the dark were wrapped in aluminium foil during incubation.

Antimicrobial effects were determined by means of viable cell counts as follows: 10 µL aliquots of the samples were taken at the beginning of the incubation and after 18 h. The diluted bacterial suspensions were transferred to MHA and incubated overnight. A 2.5 mg/mL solution of MTT (Sigma Aldrich, Steinheim, Germany) was used to facilitate the visualization of the bacterial colonies which were subsequently counted. Cells counts were plotted on a logarithmic scale using GraphPad Prism 5.01 (GraphPad Software Inc., La Jolla, CA, USA).

All dilutions were done in duplicate per experiment and all tests were conducted independently three times. Ampicillin (32 µg/mL, AppliChem, Darmstadt, Germany) served as a positive control, while MHB without the addition of samples served as negative controls. Non-inoculated samples were used as sterility controls.

### Effects of iron oxides on breeding success (hypothesis 3)

To assess whether iron oxides have any influence on the reproductive success of Bearded Vultures (Arlettaz et al., 2002), we randomly selected five breeding pairs with very pale feather colours and six pairs with very intense colours, and compared their breeding success (the total number of chicks fledged divided by the number of pairs with hatched clutches), obtaining data from 122 breeding attempts (from 1990–2006). Using telescopes, we categorised the observed neck and ventral plumage colour of individual breeding Bearded Vultures as either pale or orange. The observations were made by A.M. during a long-term breeding season monitoring programme. Pairs with paler colourations came mainly from territories located in the outer mountain ranges (average: 1,170 m, range: 900–1,450 m asl), whereas the most intensely coloured ones inhabited the axial zone of the Pyrenees (average: 1,533 m, range: 1,150–2,100 m asl). Although females are more intensely coloured than males (Negro et al., 1999) in all the territories selected, both sexes presented similar colouration to be assigned as paler or intensely coloured. The absence, or limited presence, of sources of ferruginous material in the Outer Sierras (in contrast to their much greater abundance in the axial zone, A Margalida, pers. obs., 2005) results in the paler appearance of breeding individuals there as a result of the scarcity of cosmetic soils (ochre).

*A priori*, food availability and human disturbance, two factors known to influence breeding success (Donázar, Hiraldo & Bustamante, 1993; Margalida et al., 2003; Arroyo & Razin, 2006) could be confounding factors affecting the pairs under investigation. However, recent studies quantifying the food available suggest that, in the study area, the amount of available natural food is 10-times greater than the energetic requirements needed for the breeding population (Margalida, Pérez-García & Moreno-Opo, 2017). Thus, food availability could be discarded as a factor potentially affecting breeding success. With respect to human disturbance, this factor should be more present in the Outer Sierras, affecting more intensely to territories occupied by paler individuals.

Breeding experience and the mating system (trio or pair) could also influence breeding success (Margalida et al., 2003; Carrete et al., 2006; Bertran, Margalida & Arroyo, 2009). To avoid biases related to the age of a breeding individual or its mating system (birds breeding as trios have lower breeding success than pairs; (Carrete et al., 2006), the breeding pairs selected had attempted breeding at least seven times previously, and only territories occupied by breeding pairs which had completed the incubation period were included (52–56 days; Margalida et al., 2004). To avoid pseudoreplication, we estimated the average breeding success individually in each territory and then calculated the group mean of the two categories of territory studied.

## Results

### Chemical analysis of ochre

Chemical analysis was performed on red-coloured soil (ochre) from a water bath in the Pyrenean Mountains (Fig. 2) known to be frequented by Bearded Vultures (Table 2). More than 40% of the dry matter was from iron oxides, explaining the reddish colour of the ochre soil and bath water. Other minerals were of minor occurrence.

**Table 2:** Mineral composition of reddish soil (ochre) from a water bath used by Bearded Vultures, showing the mean values of four analyses.

**Mineral**	**Content**
Fe	418 g/kg dry matter
	88.4 g/kg wet soil
Mn	1.34 g/kg dry matter
	0.18 g/kg wet soil
Cd, Co, Cr, Cu, Ni, Pb, Zn	<20 mg/kg dry matter
	<3 mg/kg wet soil

### Feather analyses: chemical composition

**Figure 3:**
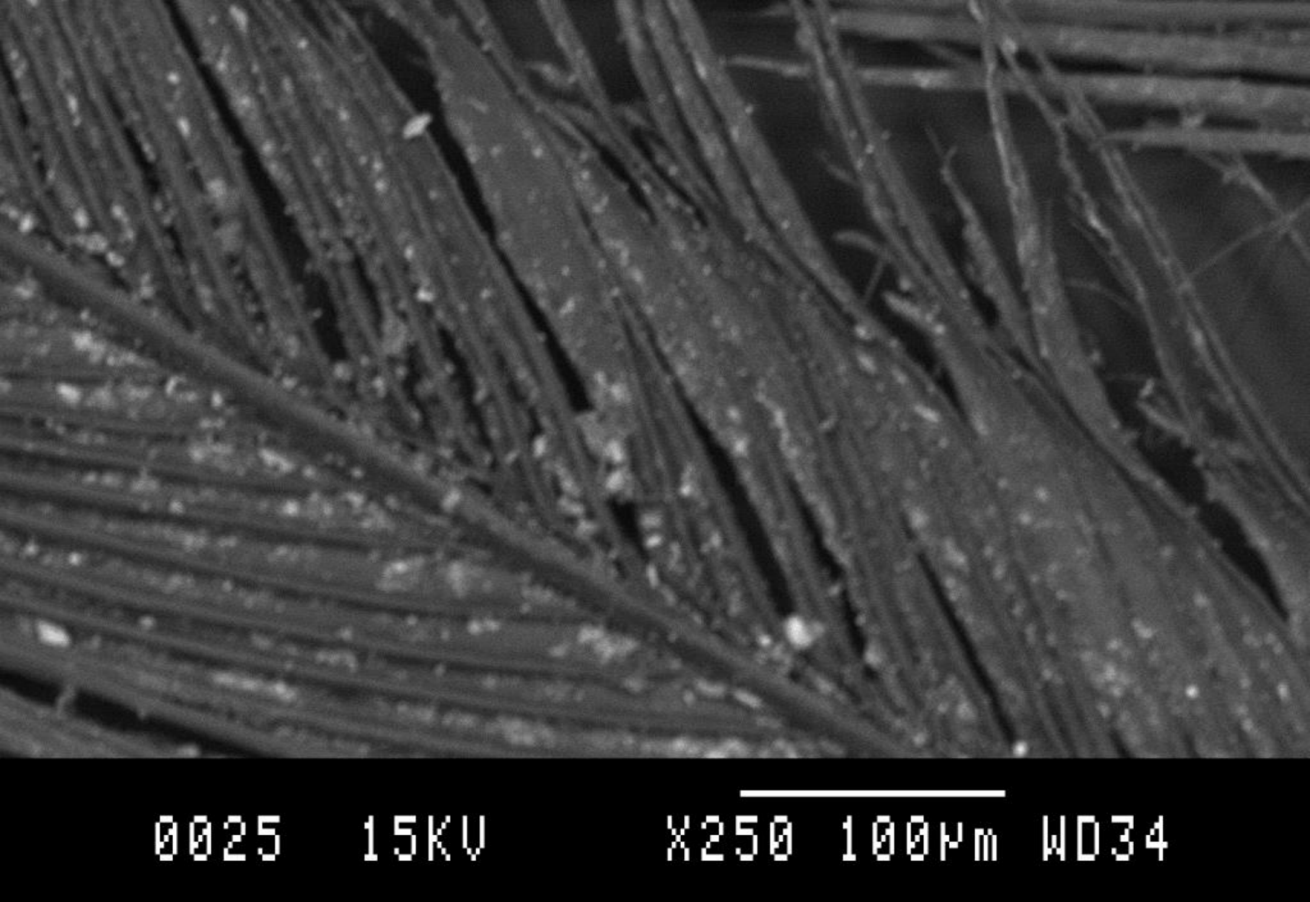
Detail of a X-ray picture carried out on an orange-coloured Bearded Vulture feather.

The results of the X-ray microanalyses performed on red-coloured Bearded Vulture feathers revealed the chemical compositions shown in Table 3. Iron was present in the feathers as goethite FeOOH-a, lepidocrocite FeOOH-g and ferrihydrite Fe(OH)_3_. Although sulphur (S) and iron (Fe) are the dominant elements, silicon (Si) and calcium (Ca) are also clearly present. The microphotograph (Fig. 3) indicates a thin homogeneous coating throughout the distal part of the down, due to normally hydrated sulfates that balance their charges with metal cations. The X-ray microanalyses at the different points of the spine, middle and distal part of the down show that while Fe, Si and S are present in minor amounts, Ca, copper (Cu), nickel (Ni), titanium (Ti), aluminium (Al) and sodium (Na) occur as the dominant elements.

**Table 3:** Chemical composition of seven red-coloured Bearded Vulture feathers obtained from three different territories. The elements obtained are listed in their relative concentrations, from high to low.

**Territory/Sample**	**Chemical composition**
Territory 13a	S, Fe, Si, Ca
Territory 13b	Fe, S, Si, Ca
Territory 14a	Fe, Si, S, Ca, Cu
Territory 14b	Fe, Si, S, Ca, Ti
Territory 14c	Fe, S, Si, Ni, Al, Na
Territory 16a	Fe, S, Si, Ca, Cu
Territory 16b	Fe, S, Si, Cu

The morphology of the feather from territory 14 shows that the coating is practically identical to that of the feather from territory 13. The feather from territory 16 had higher quantities of Fe and S, and lower quantities of Si, Ca and Cu.

### Effects of iron oxides on feather-degrading bacteria (hypothesis 2)

Feather degradation caused by *Bacillus licheniformis in vitro* was monitored daily, and no variation was detected among Bearded Vulture feathers of different basal colouration, with or without a cover of iron oxides. The feathers were fully degraded by day 7 after the inoculation of the bacilli (Table 1), as were those of eight other bird species of different colouration (including unpigmented white feathers) similarly tested by Grande, Negro & Torres (2004). Feathers from two species (black feathers from white storks, *Ciconia ciconia*, and ravens, *Corvus corax*) were fully degraded one day earlier (day 6), while those that resisted bacterial damage longest (day 8) were from the blue-crowned parakeet, *T hectocercus acuticaudata*, which contained a psittacofulvin. Therefore, the iron oxides naturally coating Bearded Vulture feathers did not significantly retard feather degradation by the particular strain of *Bacillus licheniformis* used by Grande, Negro & Torres (2004), at least *in vitro*.

### General antimicrobial effects of ochre (hypothesis 2)

**Figure 4:**
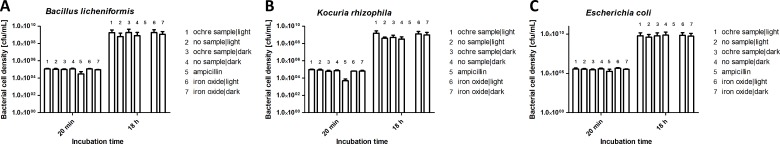
Effect of ochre (soil) (10 mg/mL) and iron oxide (water) (10 mg/mL) on the growth of *Bacillus licheniformis* (A), *Kocuria rhizophila* (B) and *Escherichia coli* (C) after 18 h incubation. The antibiotic ampillicin (32 µg/mL) was used as a positive control.

We investigated the potential antibacterial activities of ochre and iron oxides, both after incubation in the dark and in the light, against *Bacillus licheniformis* (a gram-positive bacterium), *Kocuria rhizophila* (a gram-positive bacterium from soil) and *Escherichia coli* (a common gram-negative gut bacterium of vertebrates). The growth of all three species of bacteria was unaffected either by ochre (10 mg/ mL, Fig. 4) or by iron oxides (10 mg/mL). Light conditions did not affect antibacterial activity.

### Influence of red colouration on breeding success (hypothesis 3)

**Figure 5:**
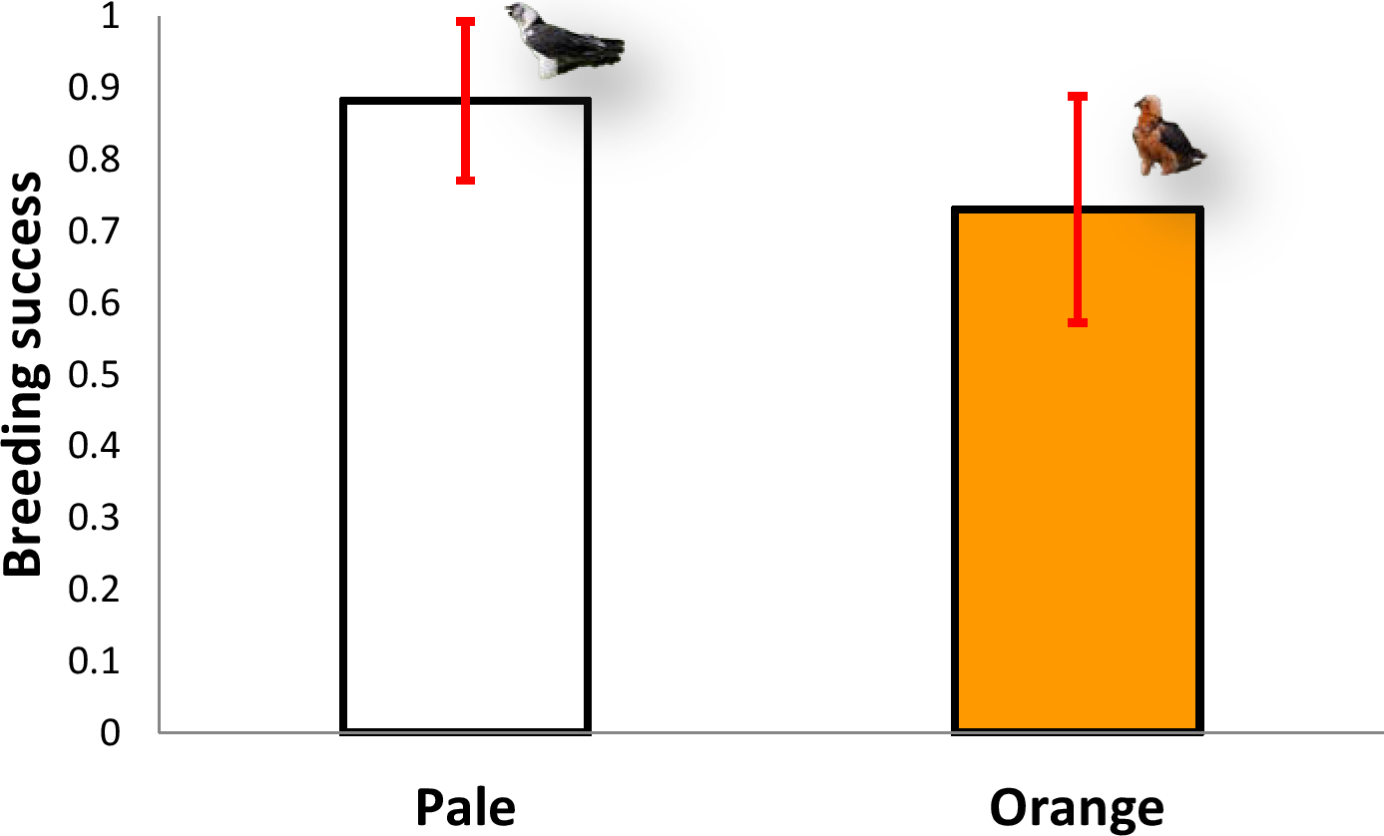
Comparison of breeding success between territories occupied by pale coloured Bearded Vultures versus orange ones. Image credit: José Antonio Sesé.

The five territories held by pale individuals (*n* = 64 breeding attempts) had a mean breeding success of 0.88 chicks/pair (range 0.7–1), excluding clutches where no eggs hatched, whereas the six territories inhabited by orange individuals (*n* = 58 breeding attempts) produced a mean of 0.76 chicks/pair (range 0.56–0.91, *n* = 6), excluding clutches where no eggs hatched (Mann–Whitney U-test, *z* = 1.67, *P* = 0.095, Fig. 5). Therefore, there were no significant differences in breeding success between the two colour categories (pale vs. orange).

## Discussion

### Chemical analysis of ochre and feathers

The orange colouration of the plumage is mostly mediated by iron oxide which is accompanied by Cu, Ni and Ti. Unlike the red soils studied in the Alps (Houston, Hall & Frey, 1993), the ochre used by Bearded Vultures in the Pyrenees is not a product of altered dolomitic limestones but of metamorphic rocks. The ferruginous waters of our study come from rocks from the Palaeozoic era. In Pyrenean ferruginous waters, recent cores of goethite coexist with other ancient minerals that have already changed to a more stable form, such as ferrihydrite. Ferruginous waters are not ubiquitous in the landscape, so that that potential Bearded Vulture bathing sites are geographically restricted (see Houston, Hall & Frey, 1993; Negro et al., 1999), and probably only some individual vultures have ready access to cosmetic soils.

### Re-evaluating the antibacterial hypothesis (hypothesis 2)

Recently, Tributsch (2016) proposed that the habit of Bearded Vultures bathing in iron-oxides might have a sanitary function (hypothesis 2). This author suggested that under visible light iron oxides kill viruses and bacteria, and convert smelly organic substances into neutral, volatile carbon dioxide gas. Ochre would therefore act as a sterilizing agent in sunlight, and provide a strategy against feather wear (Tributsch, 2016). It is noteworthy that cave-dwelling hominins occupying the same distributional ranges, and possibly the same rocky outcrops, as Bearded Vultures, both in Africa (*Homo sapiens*) and Europe (*H. neanderthalensis* and *H. sapiens*), applied ochre to themselves for at least 140,000 years (Wolf 2018). Anthropologists favour the idea that ochre application in humans was related to ritual activities, but a photoprotective action has recently been demonstrated (Rifkin et al., 2015) and other functions, such as a mosquito repellent (Rifkin, 2015) or use as a hide tanning agent have also been proposed (reviewed in Wolf et al., 2018).

The *in vitro* exposure of bearded vulture feathers to feather-degrading bacteria showed no effect of iron oxides. Degradation was not retarded in iron-coated feathers, and all tested feathers, irrespective of their basal color prior to staining degraded at exactly the same rate as the feathers of other birds. When iron oxides were applied directly to bacterial cultures, there was no perceptible inhibition of bacterial growth. Taken together, these two lines of evidence weaken the antibacterial hypothesis for ochre use in bearded vultures.

### Could iron oxides improve breeding success? (hypothesis 3)

The functional hypotheses in humans are in line with Arlettaz et al. (2002), who hypothesized that Bearded Vultures probably use iron oxides because they have pro-oxidative effects. These authors suggested that iron oxides reduce the negative effects of bacteria on egg and nestling mortality. Some experiments suggested that melanised feathers are more resistant to feather degrading bacteria than unmelanised feathers (Goldstein et al., 2004; Gunderson et al., 2008; but see Grande, Negro & Torres, 2004). However, our experiments were unable to show any antibacterial properties of ochre and iron oxides (hypothesis 2). The *in vitro* experiment with feather degrading bacteria demonstrated that iron oxides naturally coating Bearded Vulture feathers did not retard feather degradation by at least one strain of *Bacillus licheniformis*. In addition, the potential general properties of ochre and iron oxides against three bacterium species, showed no influence of ochre (10 mg/ mL) or iron oxides (10 mg/mL) on bacterial growth, after either incubation in the dark or in the light. In particular, light did not enhance any potential antibacterial activity (hypothesis 2). Natural sunlight could not be used experimentally, but we believe that the artificial light used in our experiments was a realistic substitute.

The pro-oxidation hypothesis should directly relate to the impact of egg and chick mortality caused by bacteria, and the natural absence of iron oxides should be expected to result in reduced hatching and fledging success (Arlettaz et al., 2002) (hypothesis 3). According to hypothesis 3, breeding success under similar conditions (i.e., human disturbance and food availability) should be higher in orange vulture territories. The majority (51%) of bearded vulture breeding failures take place during the hatching period (Margalida et al., 2003). Thus, the pro-oxidative effects of iron oxides should increase hatchability by reducing the negative effects of bacteria on eggs and improving nestling survival. However, our results show instead that breeding success did not differ significantly between territories occupied by orange individuals vs. pale ones, with, if anything a reverse tendency, with slightly higher average breeding success in the territories with pale individuals (with a lower frequency of visits to ferruginous water sources or a lower tendency to bathe in them) compared with orange ones. In fact, better habitat quality with respect feeding resources and lower human disturbance is present in the axial zone (Margalida, Colomer & Sanuy, 2011) with higher frequency or intensely coloured individuals with respect Outer Sierras. Thus, taking into account hypothesis 3 it should be expected that the intensely coloured individuals (with more access to ferruginous sources) inhabiting the axial area could transfer oxide particles to the eggs during the incubation process improving breeding success. Our findings do not support this hypothesis suggesting that probably breeding success can be regulated by other factors (Margalida et al., 2003; Margalida, Colomer & Oro, 2014). Therefore, the antibacterial activity hypothesis, which implies indirect effects of iron oxide on breeding success, was not supported.

### The function of orange colouration in a behavioural context (hypothesis 1)

As already mentioned, Bearded Vultures might use the orange colouration in a behavioural context (hypothesis 1; Negro et al., 1999). There are descriptions of birds exhibiting secretive behaviour when visiting water baths—bathing captive birds are extremely wary and stop bathing if they are disturbed (Houston, Hall & Frey, 1993; Frey & Roth-Callies, 1994). Anecdotal observations of wild individuals also confirm this secretive behaviour. This may be due to its behavioural function as a conspecific status signal because ochre baths are a scarce resource that would be defended against conspecifics and not revealed to them (Negro et al., 1999; Duchateau & Tellechea, 2019). The signal function of plumage colour may operate by communicating the ability of an individual to obtain limited resources (Hill, 2002; Searcy & Nowicki, 2005). In the case of Bearded Vultures, the costs involved in acquiring a bright colouration (i.e., the significant investment involved in finding the rare cosmetic soils), would allow the more orange adults to express their superiority over conspecifics. This visual signal would also indicate a good knowledge of their own territory (Negro et al., 1999). The status-signalling hypothesis (Rohwer, 1975; Searcy & Nowicki, 2005) posits that dominants benefit from their ‘badges of status’ because they reduce the number of aggressive contests in which they are involved, and subordinates benefit by avoiding interactions with superior individuals (Rohwer & Rohwer, 1978; Senar & Camerino, 1998). Accordingly, more intensively coloured individuals may be those of higher quality. However, our results do not support that more intensely coloured individuals are better breeders. This is because other confounding factors can operate on the results of reproduction (i.e., breeding experience, human disturbance, weather), being independent the higher quality status from the breeding performance. In the case of Egyptian Vultures, Van Overveld, de la Riva & Donázar (2017) suggested that the primary function of feather painting may serve pair formation and bonding and/or that it is used to show off during sexual conflicts. In fact, in bearded vultures, females are more intensely coloured than males, and in the case of polyandrous trios the subordinate male (beta male), who performed least copulations, was always the less intensively coloured individual. If feather protection was the only function of cosmetic colour, no such sex- and age-related colour differences would be expected (see Negro et al., 1999). We believe that strongly coloured individuals may be preferred as mates by individuals of the opposite sex, but that other potential explanations deserve equal consideration. In any case, as Delhey, Peters & Kempenaers (2007) pointed out, even if a protective function for the iron oxides is eventually found, “such additional functions would merely add informational content to the signal without invalidating a potential status-signalling function (Negro et al., 2002)”.

### Concluding remarks

Our findings do not support an antibacterial function for ochre application to the white feathers of Bearded Vultures (hypothesis 2), nor that incubating birds pass any protective effect of iron oxides on to their developing embryos or growing chicks (hypothesis 3). In fact, the selective application of pigments to white feather tracts—Bearded Vultures do not stain their entire plumage by rolling in the ferruginous baths—provides additional support to the hypothesis that colouring of the plumage acts as a territorial-status signal (Negro et al., 1999), although it may also benefit other behaviours including pair formation and pair-bonding, as suggested for Egyptian Vultures (Van Overveld, de la Riva & Donázar, 2017). From our findings, we conclude that the primary function of applied colouration in adult Bearded Vultures is that of status signalling at breeding sites, with a secondary function in enhancing their competitive ability to find a mate as have been proposed for greater flamingos (*Phoenicopterus roseus*) (see Amat & Rendón, 2017). Monitoring ferruginous sources using video-cameras could provide extra useful information regarding their frequency of use with respect to breeding phenology, as well as the interactions occurring there between Bearded Vultures of various age and sex classes (https://www.hbw.com/ibc/news/bearded-vulture-gypaetus-barbatus-bathing-iron-rich-mountain-springs).

##  Supplemental Information

10.7717/peerj.6783/supp-1 Dataset S1 Raw data of breeding success of bearded vultures

10.7717/peerj.6783/supp-2 Dataset S2 Effect of ochre (soil) (10 mg/mL) and iron oxide (water) (10 mg/mL) on the growth of *Bacillus licheniformis, Kocuria rhizophila* and *Escherichia coli* after 18 h incubation

10.7717/peerj.6783/supp-3 Supplemental Information 1 Permission photographs Fig. 5

## Additional Information and Declarations

### Field Study Permissions

The following information was supplied relating to field study approvals (i.e., approving body and any reference numbers): Field observations were carried out under the approval of Departament de Medi Ambient i Habitatge from Generalitat de Catalunya (project: 1992-2006).

### Data Availability

The following information was supplied regarding data availability: Raw data about bacillus experiments and breeding success are provided in the Supplemental Information.
