# Supplementary material for: Cosmetic colouring by Bearded Vultures Gypaetus barbatus: still no evidence for an antibacterial function
Source: PeerJ. 2019 May 15;7:e6783. doi: 10.7717/peerj.6783 (PMC6525594; doi:10.7717/peerj.6783)
Supplement: Dataset S1 [file peerj-07-6783-s001.docx]

**Supplementary Material**

TS1. Raw data of breeding success of bearded vultures according to the adult coloration.

| Territory | Clutches (n) | Fledglings (n) | Breeding success |  |
| --- | --- | --- | --- | --- |
| ***Pale individuals*** |  |  |  |  |
| Fl | 10 | 7 | 0.7 |  |
| Al | 11 | 10 | 0.91 |  |
| Is | 13 | 12 | 0.92 |  |
| Ve | 13 | 13 | 1 |  |
| Mo | 17 | 15 | 0.88 |  |
| **Subtotal** | **64** | **57** | **0.88** |  |
| ***Orange individuals*** |  |  |  |  |
| Bo | 9 | 4 | 0.56 |  |
| Ali | 11 | 10 | 0.91 |  |
| Ar | 11 | 7 | 0.64 |  |
| Es | 15 | 13 | 0.87 |  |
| Bon | 12 | 9 | 0.83 |  |
| Mi | 7 | 4 | 0.57 |  |
| **Subtotal** | **58** | **43** | **0.76** |  |
