# Supplementary material for: Cosmetic colouring by Bearded Vultures Gypaetus barbatus: still no evidence for an antibacterial function
Source: PeerJ. 2019 May 15;7:e6783. doi: 10.7717/peerj.6783 (PMC6525594; doi:10.7717/peerj.6783)
Supplement: Supplemental Information 1 [file peerj-07-6783-s003.docx]

# Title of PeerJ submission: Cosmetic colouring by Bearded Vultures *Gypaetus barbatus*: still no evidence for an antibacterial function

Name of copyright holder: José Antonio Sesé

Select copyrighted item: photographs | videos | other (please describe): photographs

I give my permission to PeerJ to publish my work, as described and/or appear below, under the CC-BY 4.0 license.

Signed,


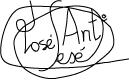


José Antonio Sesé

**Please supply the approved images(s) with the figure number(s) as they appear in the PeerJ submission**

**FIGURE 5**

**
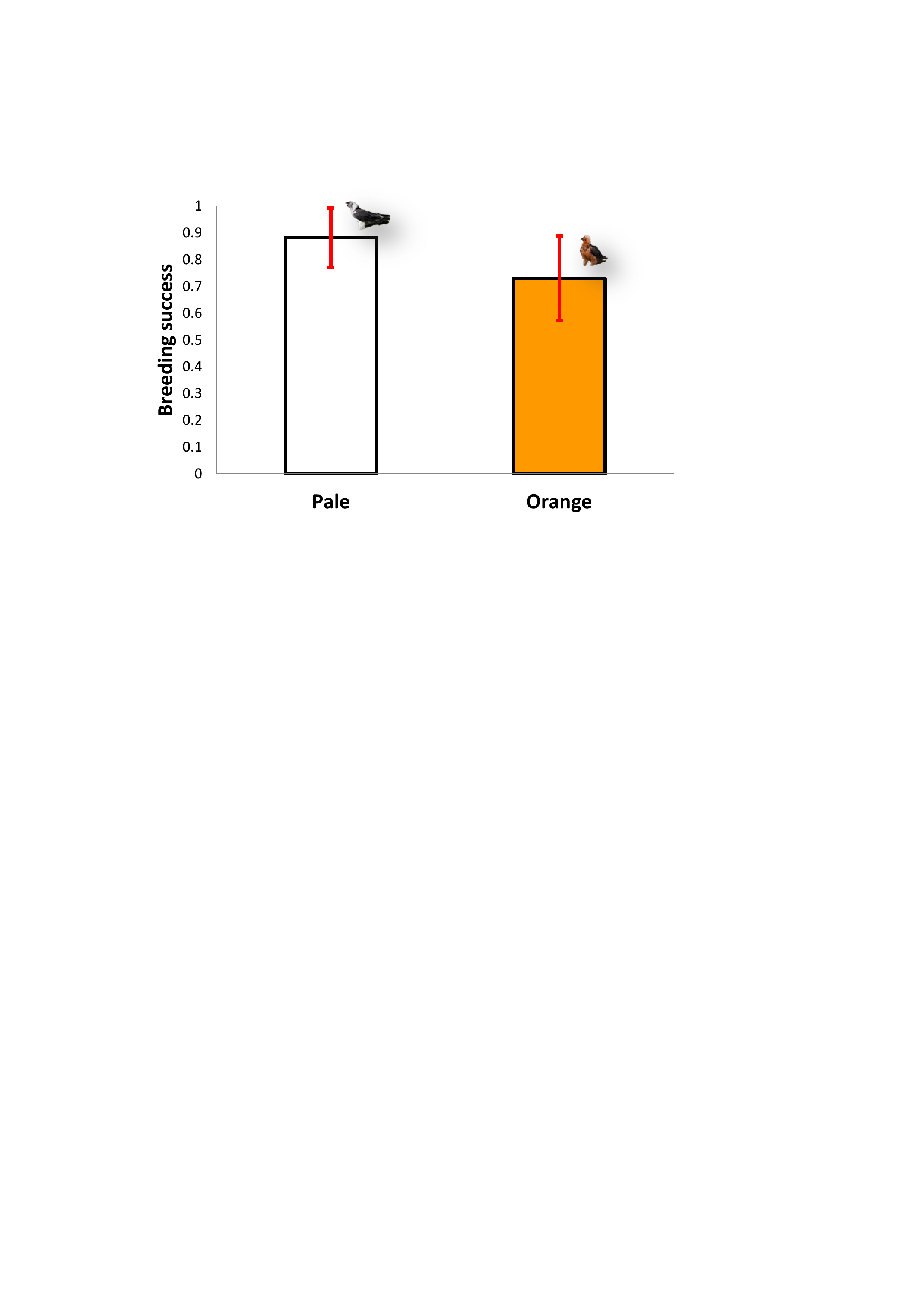
**
